# The Cues and Care Trial: A randomized controlled trial of an intervention to reduce maternal anxiety and improve developmental outcomes in very low birthweight infants

**DOI:** 10.1186/1471-2431-8-38

**Published:** 2008-09-26

**Authors:** Phyllis Zelkowitz, Nancy Feeley, Ian Shrier, Robyn Stremler, Ruta Westreich, David Dunkley, Russell Steele, Zeev Rosberger, Francine Lefebvre, Apostolos Papageorgiou

**Affiliations:** 1 Department of Psychiatry, SMBD-Jewish General Hospital, Montreal, Canada; 2 McGill University, Montreal, Canada; 3 Centre for Nursing Research, SMBD-Jewish General Hospital, Montreal, Canada; 4 School of Nursing, McGill University, Montreal, Canada; 5 Department of Epidemiology, SMBD-Jewish General Hospital, Montreal, Canada; 6 Lawrence S. Bloomberg Faculty of Nursing, University of Toronto, Toronto, Canada; 7 Department of Mathematics and Statistics, McGill University, Montreal, Canada; 8 Department of Neonatology, Hôpital Ste-Justine, Montreal, Canada; 9 Université de Montréal, Montreal, Canada; 10 Department of Neonatology, SMBD-Jewish General Hospital, Montreal, Canada

## Abstract

**Background:**

Very low birthweight infants are at risk for deficits in cognitive and language development, as well as attention and behaviour problems. Maternal sensitive behaviour (i.e. awareness of infant cues and appropriate responsiveness to those cues) in interaction with her very low birthweight infant is associated with better outcomes in these domains; however, maternal anxiety interferes with the mother's ability to interact sensitively with her very low birthweight infant. There is a need for brief, cost-effective and timely interventions that address both maternal psychological distress and interactive behaviour. The Cues and Care trial is a randomized controlled trial of an intervention designed to reduce maternal anxiety and promote sensitive interaction in mothers of very low birthweight infants.

**Methods and design:**

Mothers of singleton infants born at weights below 1500 g are recruited in the neonatal intensive care units of 2 tertiary care hospitals, and are randomly assigned to the experimental (Cues) intervention or to an attention control (Care) condition. The Cues intervention teaches mothers to attend to their own physiological, cognitive, and emotional cues that signal anxiety and worry, and to use cognitive-behavioural strategies to reduce distress. Mothers are also taught to understand infant cues and to respond sensitively to those cues. Mothers in the Care group receive general information about infant care. Both groups have 6 contacts with a trained intervener; 5 of the 6 sessions take place during the infant's hospitalization, and the sixth contact occurs after discharge, in the participant mother's home. The primary outcome is maternal symptoms of anxiety, assessed via self-report questionnaire immediately post-intervention. Secondary outcomes include maternal sensitive behaviour, maternal symptoms of posttraumatic stress, and infant development at 6 months corrected age.

**Discussion:**

The Cues and Care trial will provide important information on the efficacy of a brief, skills-based intervention to reduce anxiety and increase sensitivity in mothers of very low birthweight infants. A brief intervention of this nature may be more readily implemented as part of standard neonatal intensive care than broad-based, multi-component interventions. By intervening early, we aim to optimize developmental outcomes in these high risk infants.

**Trial Registration:**

Current Controlled Trials ISRCTN00918472

The Cues and Care Trial: A randomized controlled trial of an intervention to reduce maternal anxiety and improve developmental outcomes in very low birthweight infants

## Background

Advances in neonatal intensive care have ensured the survival of 85% of very low birthweight infants (VLBW, i.e., infants born at weights under 1500 g), but these medically fragile infants remain at high risk for developmental delays, learning difficulties, and emotional and behavioural problems. VLBW children are at twice the risk of behaviour problems compared to children in the general population [1], and are 2.5 times more likely to develop attention deficit hyperactivity disorder (ADHD) [2]. VLBW children are also at greater risk than their normal birthweight (NBW) peers for more subtle deficits including learning disabilities, anxiety disorders, social withdrawal, and social problems [2-9], and are almost 50% more likely than NBW children to require special education services [10], even in the absence of major neurocognitive disability[11]. These functional deficits persist in adolescence and early adulthood, with continued lower IQ scores and poorer academic achievement, as well as more behavioural and emotional difficulties than their NBW peers [12]. VLBW young adults are more likely to exhibit chronic health problems, lower levels of academic attainment, and higher rates of unemployment [13-16]. This high-risk group of children are disproportionately represented among users of health, special education, and social services, and tax the physical, emotional, and financial resources of their families [17,18]. In addition to direct medical costs associated with premature birth, indirect costs related to short- and long-term morbidity have been estimated at approximately $3 billion per year [19].

Given the significant risk associated with low birthweight, interventions have been designed to improve developmental outcomes in these children. These interventions have ranged from those targeting specific aspects of infant care, such as the need for supplementary sensory stimulation, and those which adapt the physical environment to the individual needs of the VLBW infant [20,21], to those offering a comprehensive package of services including medical follow-up, parent support, and educational daycare for the infants [22,23]. One significant limitation of broad, comprehensive intervention programs, such as the Infant Health and Development Program [24], is that they are of long duration, costly and time-consuming for both service providers and families. This raises questions about the economic feasibility and practical utility of transferring broad-based, multi-component interventions into clinical practice. Moreover, these programs do not always target the infants and families at greatest risk, but instead offer services to families of infants born at weights under 2500 g. Saigal and Doyle [25] have noted that one of the current challenges in the care of VLBW children is to provide support to parents to help maximize each child's potential. There is a need for brief, cost-effective and timely interventions that address specific processes that may affect infant development.

The current empirical evidence points to parental sensitivity and maternal psychological distress as two important factors influencing the development of VLBW children. Parental sensitivity includes awareness of and responsiveness to the infant's interactive cues. VLBW infants are difficult interaction partners who are less responsive to social stimulation, and produce less clear behavioural signals [26]; as a result, their caregivers may have greater difficulty in behaving sensitively with them. Not only do mothers look, smile, vocalize and touch their VLBW infants less often than mothers of NBW infants, but they are also less well able to coordinate their behaviour with their infants' brief periods of alertness [27]. Parental sensitivity supports the infant's ability to maintain attentional focus and helps to organize the infant's behaviour. This promotes cognitive and social development in VLBW infants [28], who continue to show benefits even into early adolescence in terms of social and cognitive competence [29]. In contrast, controlling, restrictive parental behaviour is associated with poorer cognitive, language, and social skills in VLBW children at 3 years of age [30]. Maternal warmth and sensitivity have been found to moderate the effects of low birth weight (LBW) on attentional deficits, hyperactivity and internalizing problems in children aged 5 to 8 years [31,32].

Psychological distress, such as feelings of anxiety, depression and posttraumatic stress, is common in mothers of VLBW infants [33,34], and can affect both parental sensitivity and infant developmental outcomes [35-37]. Such distress can persist for more than a year after the birth of the VLBW infant [38]. Moreover, VLBW infants have been found to be more reactive to maternal depression than are NBW infants [39]. The work of our research group has shown that maternal self-reported feelings of anxiety are related to less sensitive and more intrusive parenting behaviour in infancy and early childhood [40,41], and to more internalizing behaviour problems and poorer cognitive development in the infants at 24 months of age [42].

To summarize, VLBW infants are at risk for poorer cognitive and language development, as well as attention and behaviour problems. Maternal sensitive interaction with her VLBW infant is associated with better outcomes in these domains; however, maternal psychological distress interferes with the mother's ability to interact sensitively with her VLBW infant. A consensus is now emerging that early intervention programs must address both mother-child interaction patterns and maternal distress in order to have a positive impact on the mother-child relationship and child developmental outcomes [43,44]. A meta-analysis [45] of diverse interventions aimed at improving maternal sensitivity found that interventions are significantly and moderately effective in improving sensitivity in mothers of children less than 4.5 years of age (effect size Cohen's d = 0.33, p < .001). Interventions that focused only on improving sensitivity were more effective (d = .45) than all other types of interventions (d = .27) (e.g., support, or examination of mother's mental representations of the infant). Interventions using video feedback were more effective (d = .44) than those that did not use this method (d = .31). Interventions with less than 5 sessions (d = .42) were as effective as those with 5–16 sessions (d = .38), but interventions with more than 16 sessions were less effective (d = .21).

The Cues and Care trial tests a brief intervention designed to reduce anxiety and develop sensitive interaction skills among mothers of VLBW infants. By intervening at the level of maternal distress and maternal interactive behaviour, it may be possible to promote a better parenting environment and optimize child developmental outcomes. This trial addresses several gaps in the literature on interventions with low birthweight infants: 1) it targets mothers of VLBW infants, who are at greater biological risk than heavier LBW (low birthweight) infants, 2) it begins shortly after birth, before dysfunctional patterns of parent-infant interaction have been established and when brain development is most rapid [46-48], and 3) it compares the intervention to an attention control condition, thus controlling for the effects of the extra support received by mothers in the experimental condition. Moreover, the experimental intervention 1) is relatively brief compared to many previous broad-based, multi-component interventions, and so has the potential to be applied in clinical practice, 2) employs empirically-based techniques from the domains of cognitive-behaviour therapy (CBT) and parent sensitivity training, and 3) is a unique combination of 2 components – training in anxiety reduction strategies and sensitivity.

## Methods and design

The Cues and Care Trial is a randomized controlled trial with stratification by study site and centrally controlled randomization. The principal research question addressed by the Cues and Care Trial is as follows: What is the effect of a brief skills-based intervention (Cues program) on anxiety, defined as a subjective emotional state characterized by feelings of tension, apprehension, nervousness and worry [49], in mothers of VLBW infants? Anxiety is measured using the State-Trait Anxiety Inventory (STAI) [50]. The Cues intervention teaches mothers to attend to their own physiological, cognitive, and emotional cues that signal anxiety and worry, and to use cognitive-behavioural strategies to reduce distress. Mothers are also taught to understand infant cues and to respond sensitively to those cues. The primary hypothesis of the study is that immediately post-intervention, mothers in the intervention group will be less anxious than those in the attention control group. Secondary hypotheses examine differences between the two groups on other measures of maternal distress, including symptoms of posttraumatic stress, stress associated with the Neonatal Intensive Care Unit (NICU) experience, and maternal sensitivity in interaction with her infant. We also collect exploratory data on postpartum depression and on infant development at 6 months corrected age.

### Ethical considerations

The study protocol has been approved by the institutional review boards of the two hospitals where the trial is being conducted. Written informed consent is obtained from all participants prior to enrolment in the trial.

### Participants

Mothers are recruited from the NICUs at 2 Montreal hospitals, with eligible admissions totalling approximately 250 per year. Inclusion criteria are: 1) singleton infant born weighing less than 1500 grams, and mother 2) is able to speak and read either English or French, 3) is able to sign informed consent, and 4) resides within a 90-minute drive of the hospital. Mothers are excluded if: 1) they will not be caring for the infant after discharge from hospital (i.e., foster placement, adoption), 2) their infant is in a highly unstable medical condition that is likely to result in death, has a major congenital anomaly or is known to have a major sensory handicap (i.e., blind or deaf), 3) their infant is likely to be transferred or discharged in less than 4 weeks, or 4) they have given birth to multiples (twins or triplets). Mothers of multiples are excluded because pilot data showed that time constraints resulting from differing discharge dates for their infants made it difficult for them to complete the planned intervention [51]. At one site, the practice of room assignments necessitated the exclusion of mothers who shared a room with women who had already been recruited as study participants, in order to avoid contamination.

Recruitment is undertaken by a trained member of the research staff. Birth records at each site are examined several times per week for eligible new admissions. Eligible mothers are informed about the study soon after birth as most require several weeks to decide whether to participate. At the initial contact, the recruiter gives the mother written information about the study and a copy of the consent form. Mothers are encouraged to make a decision by the 4^th ^week after birth, in order to allow sufficient time to participate in the sessions prior to discharge.

Randomization is centrally controlled, concealed, in random blocks of 4 or 6, and stratified by hospital (stratification is required because one hospital serves a primarily francophone population while the other has a more diverse, multi-ethnic patient group). Randomization takes place using *randomize.net *, a centrally controlled, secure, online service.

Power analysis was based on the work of Mohide [52], which indicated that a change of 7 points on the STAI scale (the primary outcome) is considered clinically meaningful. Setting alpha = 0.05 and power = 0.8, we would need 40 subjects per group [53]. To account for attrition, a sample of 46 mothers per group will be recruited.

### Trial interventions

The experimental "Cues" intervention consists of 6 sessions to teach mothers to: 1) read their own cues and recognize signs of anxiety/distress, 2) utilize various strategies to reduce their distress, including muscle relaxation, imagery, and cognitive reframing, 3) read their infant's communication cues, and 4) respond sensitively to infant cues and distress. In the first two sessions, the intervener explains the relationship between thoughts, feelings, and behaviour, and teaches mothers how to identify negative automatic thoughts. Participants acquire skills that help them to relax and to counteract maladaptive thought patterns. The next two sessions focus on understanding the behaviour of VLBW infants, identifying infant states cues and learning how to interact sensitively with the infant. The fifth session is devoted to mother-infant interaction during feeding. Each teaching session lasts 60 – 90 minutes. There is also a telephone follow-up call, to review the techniques that have been taught and to maintain contact with participant mothers. The first 5 sessions take place in the NICU at a frequency of 1–2 sessions per week. The last session takes place in the mother's home 2–4 weeks after discharge. Total dose is 9–10 hours.

The program is delivered in one-on-one sessions between the mother and an intervener. Group sessions are not feasible because mothers differ greatly in the timing and frequency of their NICU visits; moreover, other studies with postpartum women report a high level of non-compliance with group interventions [54]. Prior to each session, the mother is given a booklet describing the main ideas to be discussed at that session, and is asked to read the booklet before meeting with the intervener. Learning exercises are also included to assist mothers to apply the content to their own experience.

The final session takes place 2–4 weeks after discharge in the mother's home, as this is a time of heightened anxiety when the mother first assumes full responsibility for infant care. The convenience of a home visit facilitates mothers' compliance. We videotape the mother playing with her infant for 10 minutes. The intervener and mother then review the tape to help the mother to apply the skills acquired in previous sessions. Anxiety reduction techniques are also reviewed. This modality was incorporated in the Cues intervention because a meta-analysis of interventions designed to enhance maternal sensitivity in mothers of both preterm and full-term infants has shown that interventions that used video feedback were more effective than those that did not use this method [45].

The trial includes an attention control or "Care" group, in order to: 1) control for effects of the additional support and contact that experimental mothers receive, 2) promote participation by providing something beyond usual care for all participants, and 3) minimize drop-out from the control group. Control group mothers also have 6 contacts with an intervener at regular intervals coinciding with the timing of the experimental group contacts. The format for these contacts parallels that of the intervention group. Prior to each contact, the mother is given a brochure. Mothers are given information on common health problems of preterm infants as well as general information about infant care and feeding readily available to all mothers of infants. At each contact, the control group intervener meets with the mother in a private setting, discusses the content with the mother and answers any questions.

Both groups receive usual medical, nursing and other care provided at the two study sites. We collect data on other services that mothers in both groups receive, such as consultations with mental health professionals or participation in support groups, at enrolment, prior to NICU discharge and post-intervention.

The interveners are nurses or clinical psychology graduate students, who are skilled in developing therapeutic relationships and teaching. To avoid contamination, some interveners are trained to deliver the Cues intervention, and other interveners are trained to deliver the Care intervention. Each participant mother is assigned to one intervener, who delivers all intervention sessions to that mother.

Manuals have been developed for both the Cues and the Care interventions; these manuals explicitly describe the teaching content and methods for each session. During their training, interveners read the manual and review the purpose, content and methods of each session with the principal investigators. In addition, interveners delivering the Cues program are trained with the *Keys to Caregiving *program [55] developed to train professionals how to teach parents about sensitive interaction. This training program consists of a 5 1/2 hour videotape with an accompanying 70-page manual. Interveners view the tapes, read the accompanying manual and discuss the content. After completing the *Keys Program*, the interveners also view videotapes of mother-infant interactions to verify their understanding of the content. Training for the anxiety-reduction components of the Cues intervention is done by experienced cognitive-behaviour therapists on the research team. First, interveners read and discuss articles on cognitive-behavioural interventions in treating anxiety symptoms. Second, role play is used to practice specific intervention techniques. Third, under supervision each intervener practices intervention skills with a hospitalized postpartum mother (not a study participant).

Training of the control group interveners delivering the Care program is done by a nurse/collaborator. The control group interveners do not have prior knowledge, nor are they taught the cognitive-behavioural interventions or the sensitivity content. They are instructed to refer mothers with any concerns beyond the scope of the topics addressed in the booklets to the appropriate NICU staff member (i.e., physician, social worker).

To ensure consistency of delivery across interveners, the interveners refer to their respective manuals and follow the procedures outlined in them. Interveners also record the relevant details of each session with each mother on a standardized record sheet. There is ongoing supervision during the course of the study, with separate meetings for the experimental and control group interveners. Furthermore, every *7*^*th *^session (15% of total) is audio-taped and reviewed by the supervisors and feedback given to the intervener involved to ensure fidelity to the intervention design.

### Primary and secondary outcomes

The primary outcome of the trial is maternal anxiety, measured by the state portion of the State-Trait Anxiety Inventory (STAI) [50]. This self-report measure consists of 20 items that ask about the intensity of the respondent's feelings of anxiety, tension, apprehension, nervousness and worry at the time of completion of the questionnaire. The STAI has been translated into more than 60 languages and has been used in thousands of studies designed to assess stress-related psychiatric and medical disorders, and in treatment outcome studies [49]. Respondents rate each item on a 4-point scale, indicating whether they experience the particular feeling: 1 (not at all), 2 (somewhat), 3 (moderately so), or 4 (very much so). Standardized on more than 5000 subjects, the STAI has high internal consistency, with a median Cronbach's alpha of .93 (in VLBW mothers, Cronbach's alpha = 0.91). In terms of construct validity, STAI scores increase significantly in stressful situations, and decrease after relaxation training.

The following are secondary outcome measures:

1. Maternal perinatal posttraumatic stress is assessed with the *Perinatal PTSD Questionnaire *[56]*(PPQ)*. This self-report questionnaire assesses symptoms of posttraumatic stress specifically related to the childbirth experience. Fourteen items tap intrusive thoughts, avoidance and increased arousal. Internal consistency of the measure is high (alpha = .83) and test-retest reliability very good (r = .92). PPQ scores have been significantly correlated with other well-validated measures of posttraumatic stress [57], and with use of psychotherapy [58].

2. The revised Parental Stress Scale: Neonatal Intensive Care Unit (PSS: NICU) [59] is used to assess two dimensions of stress particularly salient to the experience of mothers of hospitalized VLBW infants: a) stress related to the infant's behaviour and appearance, and b) stress related to feeling restricted in their maternal or caregiver role. The internal consistency of this self-report questionnaire is excellent (Cronbach alpha's .89–.94). Evidence of construct validity has been demonstrated, in that the PSS: NICU has been significantly correlated with state anxiety scores (r = .45).

3. Sensitivity of mother-infant interaction is assessed immediately post-intervention and at the 6-month follow-up with the Global Rating Scales of Mother-Infant Interaction [60]. A five-minute videotape of mother-infant interaction is made in the home; mothers are instructed to play with their infants without the use of toys. Maternal and infant behaviours are then rated, by a coder who is blind to the mother's group assignment, on several dimensions, including maternal sensitivity, intrusiveness, remoteness, and depression, as well as infant engagement, liveliness and fretfulness. The overall quality of the interaction between mother and infant is also rated. We evaluate inter-rater reliability by having two coders rate the videotapes, and then comparing their scores using intraclass correlations. The measure has been found to discriminate between mothers with and without postpartum depression, and has also been used with other clinical groups such as schizophrenic mothers and mothers with borderline personality disorder [61].

### Additional variables

For the purposes of sample description, we collect data on demographic variables such as maternal age, education, occupation, parity, and current use of psychosocial services (e.g., social worker, support groups). Infant illness severity is assessed with the Revised Nursery Neurobiologic Risk Score (NBRS) [62]. It includes 7 items that assess the presence, severity and duration of medical events associated with the risk of later abnormal neurodevelopment for the infant. The NBRS is scored following a review of the infant's medical record. NBRS scores correlate with later child neurological examination scores [63], as well as with poorer subsequent social, cognitive and psychomotor development of children born VLBW [64-66]. At the time of discharge, the NBRS is scored by a rater blind to the mother's group assignment.

We are also collecting data for exploratory analyses of two important outcome variables:

1. Maternal depression is assessed using the Edinburgh Postnatal Depression Scale (EPDS)[67]. This 10-item scale is designed to screen for postpartum depression in community samples. The items are rated on a scale from 0 to 3, and refer to symptoms experienced in the past 7 days. Respondents who score above the cut point of 12 are classified as at risk for a depressive disorder. The split-half reliability of the scale is 0.88, and the standardized alpha coefficient is 0.87. In validation studies, using a cut score of 12, the EPDS was found to have a sensitivity of 68% to 95%, and a specificity of 78% to 96%, when compared to a diagnosis of major depression made by psychiatric interview [68-70].

2. Bayley Scales of Infant Development, 3^rd ^edition (BSID-III, 2005) [71] are used to gather pilot data on infant cognitive development. This is the most recent revision of a widely used measure of infant development, and consists of 5 scales: cognitive, language, motor, social-emotional, and adaptive. This instrument has excellent psychometric properties, and has been validated on over 1700 children.

Given that maternal depression is a dichotomous outcome, the sample size required to adequately test any related hypotheses would be prohibitive. Similarly, the trial is not adequately powered to test differences between the experimental and control groups in terms of infant developmental outcome on the BSID-III. Although we cannot test hypotheses related to these variables, the pilot data that we are collecting on these variables will help with future projects in this area.

### Procedures

**Figure 1:**
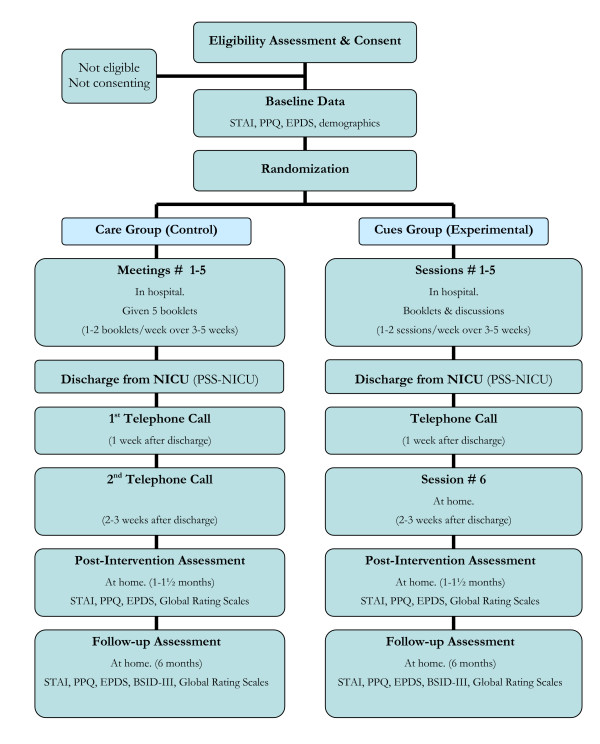
**Cues and Care Trial timeline**. STAI = State TraitAnxiety Inventory. EPDS = Edinburgh Postnatal Depression Scale. PPQ = Perinatal PTSD Questionnaire. PSS-NICU = Parent Stressor Scale: Neonatal Intensive Care Unit. BSID-III = Bayley Scales of Infant Development-third edition.

The study timeline can be found in Figure 1. Following recruitment, informed consent is obtained, and baseline measures (STAI, PPQ, and EPDS) are administered. Participants are then randomized to either the Cues or the Care condition. Within a day of randomization, an intervener contacts the participant and arranges for the first session of the intervention.

Post-intervention measures are administered at the participant's home 2 – 3 weeks after the intervention ends (when infants are 1 – 1.5 months corrected age). This assessment includes observation of mother-infant interaction, and completion of the self-report measures of anxiety, depression, and posttraumatic stress. In addition, participant mothers are asked to report on their satisfaction with the intervention that they received. A follow-up assessment of mothers and infants takes place at home when the infants are 6 months old (corrected age). At this time, the BSID-III is administered, in conjunction with observation of mother-infant interaction, and the completion of the same set of self-report measures used at baseline and at the immediate post-intervention assessment.

### Blinding

It is not possible to blind participant mothers because the experimental treatment condition teaches mothers specific skills to reduce distress and interact sensitively with their infants, while mothers in the control group receive non-specific attention. However, we tell both groups that their intervention *may *reduce anxiety and distress, in order to minimize any differences in participants' expectations.

Careful attention is paid to the blinding of assessors and data collectors. All pre-intervention data are obtained blinded because they are collected prior to randomization. The research assistants who collect the post-intervention data (1 & 6 months of age) are not involved in the pre-intervention data collection. Research assistants are instructed not to ask participants about their group assignment, and participants are asked not to reveal their group assignment to research or medical staff. Mothers read and respond to self-report questionnaires independently. The research assistants who videotape interactions in the home are not involved in the coding of the videotapes. All interaction videotapes are coded at a later date by a research assistant hired just for this task, and who has no contact with the NICU or with participants and thus is blind to group assignment. Other staff, also blind to group assignment, are trained to score the NBRS via chart review. Finally, post-intervention research assistants and interveners are housed in separate buildings to minimize the chance of information transfer, and there are separate meetings for interveners and other research staff.

### Contamination

Contamination is a potential concern when delivering an intervention to mothers in NICUs, which are large open units, with infant incubators located in close proximity to one another. Inevitably, there is interaction between mothers. In order to address this issue, we have adopted inclusion/exclusion criteria that preclude the recruitment of mothers who are sharing a room. Once participants are enrolled in the study, we employ several strategies to deal with contamination. First, we explain to participants that we can only test the effectiveness of the intervention if they do not share information. Second, interveners are instructed not to discuss the program content with non-participants or with NICU staff. Third, whenever possible, interveners deliver the intervention sessions at a private location where their discussion cannot be overheard by other mothers or NICU staff. Fourth, when sessions take place at the infant's bedside, the interveners place a screen around the bedside to maximize privacy. Fifth, when the intervention is completed we ask mothers if they have shared any of the program information with other NICU mothers, and if so with whom. Finally, we measure contamination post-intervention by asking mothers in both the experimental and control groups to respond to a questionnaire testing their knowledge of the content of both programs and to indicate the source of this knowledge.

### Planned analyses

No interim analyses are planned. Upon completion of the study, we will compare baseline values for demographic variables to assess the adequacy of randomization. These include maternal age, education, parity, psychosocial services used by the mother, and infant illness severity. The primary outcome is the difference in STAI scores between the intervention group and the control group, evaluated immediately post-intervention. This is a continuous measure and will be analyzed using an unpaired t-test (i.e. comparison of scores between two independent samples) and an intent-to-treat strategy. The overall significance level will be considered at a two-sided 0.05 level. As a secondary analysis, we will use a multiple regression model to account for other predictors of outcome, including baseline STAI, and the demographic variables noted immediately above. Finally, as an exploratory analysis, we will compare groups based on actual treatment received in addition to our primary intention-to-treat analysis. The analysis of secondary measures will proceed in a likewise fashion. We will also report on contamination and describe other services used during and after the intervention. Because we only have 46 subjects per group, we cannot use sophisticated multiple imputation techniques to estimate expected results in subjects who withdraw from the study. Therefore, we will document and describe the reasons for attrition within each group, but exclude these subjects from the multiple regression analyses.

### Safety

The Cues and Care programs are psychosocial interventions, so there are no medical risks to either mother or infant. Mothers may find that the completion of questionnaires asking about anxiety and stress may arouse feelings of distress, though this has not been a problem in pilot work; those who experience such distress will be offered a consultation with one of the clinical psychologists affiliated with the trial, who may then refer them to appropriate services as needed. In addition, all mothers who score above the cut point for depression on the EPDS are offered a referral for evaluation by a mental health professional.

## Discussion

Conducting a randomized controlled trial of a psychosocial intervention in a critical care unit of a hospital poses significant challenges. Timing of recruitment is an important factor: potential participants must be approached early enough after the infant's birth to allow sufficient time to complete the intervention prior to the infant's discharge. However, mothers are unlikely to agree to participate until their infant's condition is relatively stable. Moreover, depending on the number of admissions to the NICU, stable infants are sometimes transferred to other hospitals so that their growth can be monitored before being discharged home. Such transfers may interfere with completion of the intervention program. Recruiters and interveners have developed procedures to address these issues, by delaying the recruitment of mothers of extremely small or ill neonates, and by working closely with the transfer coordinators of the NICUs.

The Cues and Care trial will provide important information on the efficacy of a brief, skills-based intervention to reduce anxiety and increase sensitivity in mothers of VLBW infants. A brief intervention of this nature may be more readily implemented as part of standard NICU care than broad-based, multi-component interventions. By intervening early, we aim to optimize developmental outcomes in this high risk group of infants.

## Abbreviations

ADHD: Attention deficit hyperactivity disorder; BSID-III: Bayley Scales of Infant Development, 3^rd ^edition; CBT: Cognitive-behaviour therapy; EPDS: Edinburgh Postnatal Depression Scale; LBW: low birthweight; MCID: Minimal clinically important difference; NBRS: Nursery Neurobiologic Risk Score; NBW: Normal birthweight; NICU: Neonatal intensive care unit; PPQ: Perinatal PTSD Questionnaire; PSS:NICU: Parental Stress Scale: Neonatal Intensive Care Unit; PTSD: Posttraumatic stress disorder; STAI: State-Trait Anxiety Inventory; VLBW: Very low birthweight.

## Competing interests

The authors declare that they have no competing interests.

## Authors' contributions

PZ and NF conceived of the study, designed the interventions, and share responsibility for the conduct of the trial. RobynS advised on trial design, methodology, and recruitment and retention of participants. IS contributed to the design of the study, methodology, and supervises data management. RW and DD designed the cognitive-behavioural components of the Cues intervention, and trained and supervise the interveners. ZR advised on the design and conduct of trials of psychosocial interventions. AP and FL act as the liaisons with the medical staff at two sites, and supervise the collection of medical data. RussellS contributed to the design of the study, methodology, and will supervise data analysis. All authors read and approved the final manuscript.

## Pre-publication history

The pre-publication history for this paper can be accessed here:



## References

[B1] Gray RF, Indurkhya A, McCormick MC (2004). Prevalence, stability, and predictors of clinically significant behavior problems in low birth weight children at 3, 5, and 8 years of age. Pediatrics.

[B2] Bhutta AT, Cleves MA, Casey PH, Cradock MM, Anand KJS (2002). Cognitive and behavioral outcomes of school-aged children who were born preterm: A meta-analysis. JAMA.

[B3] Breslau N, Chilcoat H (2000). Psychiatric sequelae of low birth weight at 11 years. Biological Psychiatry.

[B4] Hille ETM, den Ouden AL, Saigal S, Wolke D, Lambert M, Whitaker A (2001). Behavioral problems in children who weigh 1000 grams or less at birth in four countries. The Lancet.

[B5] Horwood LJ, Mogridge N, Darlow BA (1998). Cognitive, educational, and behavioural outcomes at 7 to 8 years in a national very low birthweight cohort. Arch Dis Child Fetal Neonatal Ed.

[B6] Nadeau L, Tessier R, Boivin M, Lefebvre F, Robaey P (2003). Extremely premature and very low birthweight infants: A double hazard population?. Social Development.

[B7] Schothorst P, van Engeland H (1996). Long-term behavioral sequelae of prematurity. Journal of the American Academy of Child & Adolescent Psychiatry.

[B8] Whitaker A, Van Rossem R, Feldman JF, Schonfeld IS, Pinto-Martin JA, Tore C (1997). Psychiatric outcomes in low-birth-weight children at 6 years: Relation to neonatal cranial ultrasound abnormalities. Arch Gen Psychiat.

[B9] Zelkowitz P, Papageorgiou A, Zelazo PR, Salomon Weiss MJ (1995). Behavioral adjustment in very low and normal birth weight children. Journal of Clinical Child Psychology.

[B10] Chaikind S, Corman H (1991). The impact of low birthweight on special education costs. Journal of Health Economics.

[B11] Wilson-Costello D (2007). Is there evidence that long-term outcomes have improved with intensive care?. Semin Fetal Neonatal Med.

[B12] Saigal S (2000). Follow-up of very low birthweight babies to adolescence. Seminars in Neonatology.

[B13] Hack M, Flannery DJ, Schluchter M, Cartar L, Borawski E, Klein N (2002). Outcomes in young adulthood for very-low-birth-weight infants. New England Journal of Medicine.

[B14] Mazurier E, Lefebvre F, Tessier R (1999). Educational achievement and intelligence at 16–21 years of ex-prematures born at < 1000 grams. Pediatric Research.

[B15] Tideman E, Ley D, Bjerre I, Forslund M (2001). Longitudinal follow-up of children born preterm: Somatic and mental health, self-esteem and quality of life at age 19. Early Human Development.

[B16] Kristensen P, Bjerkedal T, Irgens LM (2004). Birthweight and work participation in adulthood. Int J Epidemiol.

[B17] Petrou S (2003). Economic consequences of preterm birth and low birthweight. BJOG.

[B18] Petrou S, Sach T, Davidson L (2001). The long-term costs of preterm birth and low birth weight: Results of a systematic review. Child Care Health Dev.

[B19] Health Canada (2002). Economic burden of illness in Canada, 1998.

[B20] Als H, Lawhon G, Duffy FH, McAnulty GB, Gibes-Grossman R, Blickman JG (1994). Individualized developmental care for the very low birth-weight preterm infant:medical and neurofunctional effects. JAMA.

[B21] Stevens B, Petryshen P, Hawkins J, Smith B, Taylor P (1996). Developmental versus conventional care: A comparison of clinical outcomes for very low birth weight infants. Canadian Journal of Nursing Research.

[B22] Hernandez-Reif M, Field TM, Osofsky JD, Fitzgerald HE (2000). Preterm infants benefit from early interventions. Handbook of infant mental health.

[B23] Hess CR, Teti DM (2005). NICU-based interventions for high-risk infants. Handbook of research methods in developmental science.

[B24] Brooks-Gunn J, McCarton C, Casey PH, McCormick MC, Bauer CR, Bernbaum JC (1994). Early intervention in low-birth-weight premature infants: Results through age 5 years from the Infant Health Development Program. JAMA.

[B25] Saigal S, Doyle LW (2008). An overview of mortality and sequelae of preterm birth from infancy to adulthood. Lancet.

[B26] Singer LT, Fulton S, Davillier M, Koshy D, Salvator A, Baley J (2003). Effects of infant risk status and maternal psychological distress on maternal-infant interactions during the first year of life. J Dev Behav Pediatr.

[B27] Feldman R, Eidelman A (2007). Maternal postpartum behavior and the emergence of infant-mother and infant-father synchrony in preterm and full-term infants: The role of neonatal vagal tone. Developmental Psychobiology.

[B28] Landry SH, Garner PW, Swank P, Baldwin CD (1996). Effects of maternal scaffolding during joint toy play with preterm and full-term infants. Merrill-Palmer Quarterly.

[B29] Beckwith L, Rodning C, Cohen S (1992). Preterm children at early adolescence and continuity and discontinuity in maternal responsiveness from infancy. Child Development.

[B30] Landry SH, Smith KE, Miller-Loncar CL, Swank PR (1997). Responsiveness and initiative: Two aspects of social competence. Infant Behavior & Development.

[B31] Laucht M, Esser G, Schmidt M (2001). Differential development of infants at risk for psychopathology: the moderating role of early maternal responsivity. Dev Med Child Neurol.

[B32] Tully LA, Arseneault L, Caspi A, Moffitt TE, Morgan J (2004). Does maternal warmth moderate the effects of birth weight on twins' attention-deficit/hyperactivity disorder (ADHD) symptoms and low IQ?. J Consult Clin Psychol.

[B33] Miles MS, Holditch-Davis D, Schwartz TA, Scher M (2007). Depressive symptoms in mothers of prematurely born infants. J Dev Behav Pediatr.

[B34] Singer L, Salvator A, Guo S, Collin M, Lilien L, Baley J (1999). Maternal psychological distress and parenting stress after the birth of a very low-birth-weight infant. JAMA.

[B35] Assel MA, Landry SH, Swank PR, Steelman L, Miller-Loncar C, Smith KE (2002). How do mothers' childrearing histories, stress and parenting affect children's behavioural outcomes?. Child Care Health Dev.

[B36] Pierrehumbert B, Nicole A, Muller-Nix C, Forcada-Guex M, Ansermet F (2003). Parental post-traumatic reactions after premature birth: Implications for sleeping and eating problems in the infant. Archives of Disease in Childhood: Fetal and Neonatal Edition.

[B37] Karatzias T, Chouliara Z, Maxton F, Freer Y, Power K (2007). Post-traumatic symptomatology in parents with premature infants: A systematic review of the literature. Journal of Prenatal and Perinatal Psychology and Health.

[B38] Kersting A, Dorsch M, Wesselmann U, Ludorff K, Witthaut J, Ohrmann P (2004). Maternal posttraumatic stress response after the birth of a very low-birth-weight infant. Journal of Psychosomatic Research.

[B39] Bugental DB, Beaulieu D, Schwartz A (2008). Hormonal sensitivity of preterm versus full-term infants to the effects of maternal depression. Infant Behav Dev.

[B40] Feeley N, Gottlieb L, Zelkowitz P (2005). Infant, mother and contextual predictors of mother-very low birth weight infant interaction at 9 months. J Dev Behav Pediatr.

[B41] Zelkowitz P, Papageorgiou A, Bardin C, Wang T (2008). Persistent maternal anxiety affects the interaction between mothers and their very low birthweight children at 24 months.

[B42] Zelkowitz P, Bardin C, Papageorgiou A (2003). Maternal anxiety and behavior in the NICU and impact on development of infants born < 1500 g (VLBW). Pediatric Research.

[B43] Forman DR, O'Hara MW, Stuart S, Gorman L, Larsen KE, Coy KC (2007). Effective treatment for postpartum depression is not sufficient to improve the developing mother-child relationship. Development and Psychopathology.

[B44] Nylen KJ, Moran TE, Franklin CL, O'Hara MW (2006). Maternal depression: A review of relevant treatment approaches for mothers and infants. Infant Mental Health Journal.

[B45] Bakermans-Kranenburg MJ, van IJzendoorn MH, Juffer F (2003). Less is more: Meta-analyses of sensitivity and attachment interventions in early childhood. Psychological Bulletin.

[B46] Gunnar MR (2003). Integrating Neuroscience and Psychological Approaches in the Study of Early Experiences. Ann NY Acad Sci.

[B47] Mustard F (2004). Towards an integrated system of early childhood development programs.

[B48] Siegel DJ (1999). The developing mind: Toward a neurobiology of interpersonal experience.

[B49] Spielberger CD, Reheiser EC, Owen AE, Sydeman SJ, Maruish ME (2004). Measuring the psychological vital signs of anxiety, anger, depression, and curiosity in treatment planning and outcomes assessment. The use of psychological testing for treatment planning and outcomes assessment.

[B50] Spielberger CD, Gorsuch RL, Lushene R, Vagg PR, Jacobs GA (1983). State-Trait Anxiety Inventory for Adults.

[B51] Feeley N, Zelkowitz P, Charbonneau L, Cormier C, Lacroix A, Ste-Marie C (2008). Promoting mothers' ability to interact sensitively with their very-low birthweight infant: A pilot study. Advances in Neonatal Nursing.

[B52] Mohide A, Tudiver F, Bass MJ, Dunn EV, Norton PG, Stewart M (1992). Evaluating the effectiveness of a multifaceted community-based support program. Assessing interventions: Traditional and innovative methods.

[B53] Dupont WD, Plummer WD (1997). *PS *power and sample size program. Controlled Clinical Trials.

[B54] Dennis C-L, Creedy D (2004). Psychosocial and psychological interventions for preventing postpartum depressionn. Cochrane Database Syst Rev.

[B55] Sumner G, Barnard K (2003). Keys to Caregiving: A study guide.

[B56] DeMier RL, Hynan MT, Harris HB, Manniello RL (1996). Perinatal stressors as predictors of symptoms of posttraumatic stress in mothers of infants at high risk. Journal of Perinatology.

[B57] Quinnell FA, Hynan MT (1999). Convergent and Discriminant Validity of the Perinatal PTSD Questionnaire (PPQ): A Preliminary Study. Journal of Traumatic Stress.

[B58] Callahan JL, Borja SE, Hynan MT (2006). Modification of the Perinatal PTSD Questionnaire to enhance clinical utility. J Perinatol.

[B59] Miles MS, Funk SG, Carlson J (1993). Parental Stressor Scale: Neonatal intensive care unit. Nursing Research.

[B60] Murray L, Fiori-Cowley A, Hooper R, Cooper P (1996). The impact of postnatal depression and associated adversity on early mother-infant interactions and later infant outcome. Child Development.

[B61] Gunning M, Conroy S, Valoriani V, Figueiredo B, Kammerer MH, Muzik M (2004). Measurement of mother-infant interactions and the home environment in a European setting: preliminary results from a cross-cultural study. Br J Psychiatry Suppl.

[B62] Brazy JE, Goldstein RF, Oehler JM, Gustafson KE, Thompson RJ (1993). Nursery Neurobiological Risk Score: Levels of risk and relationships with nonmedical factors. J Dev Behav Pediatr.

[B63] Brazy JE, Eckerman CO, Oehler JM, Goldstein RF, O'Rand AM (1991). Nursery Neurobiologic Risk Score: Important factors in predicting outcome in very low birth weight infants. Journal of Pediatrics.

[B64] Lefebvre F, Gregoire MC, Dubois J, Glorieux J (1998). Nursery neurobiologic risk score and outcome at 18 months. Acta Paediatr.

[B65] Luciana M, Lindeke L, Georgieff M, Mills M, Nelson CA (1999). Neurobehavioral evidence for working-memory deficits in school-age children with histories of prematurity. Developmental Medicine & Child Neurology.

[B66] Thompson RJ, Gustafson KE, Oehler JM, Catlett AT, Brazy JE, Goldstein RF (1997). Developmental outcome of very low birth weight infants at four years of age as a function of biological risk and psychosocial risk. J Dev Behav Pediatr.

[B67] Cox JL, Chapman G, Murray D, Jones P (1996). Validation of the Edinburgh Postnatal Depression Scale (EPDS) in non-postnatal women. Journal of Affective Disorders.

[B68] Cox JL, Holden JM, Sagovsky R (1987). Detection of postnatal depression: Development of the 10-item Edinburgh Postnatal Depression Scale. British Journal of Psychiatry.

[B69] Murray L, Carothers AD (1990). The validation of the Edinburgh Post-natal Depression Scale on a community sample. British Journal of Psychiatry.

[B70] Zelkowitz P, Milet TH (1995). Screening for post-partum depression in a community sample. Can J Psychiatry.

[B71] Bayley N (2005). Bayley Scales of Infant and Toddler Development.

